# A High-Quality Sample Generation Method for Improving Steel Surface Defect Inspection

**DOI:** 10.3390/s24082642

**Published:** 2024-04-20

**Authors:** Yu He, Shuai Li, Xin Wen, Jing Xu

**Affiliations:** 1 Department of Software Engineering, Shenyang University of Technology, Shenyang 110870, China; ls010221@163.com (S.L.); wen_xin@sut.edu.cn (X.W.); 2 Department of Mechanical Engineering, Shenyang University of Technology, Shenyang 110870, China; jing_xu@sut.edu.cn

**Keywords:** defect sample generation, generative adversarial network (GAN), production-and-elimination, defect inspection

## Abstract

Defect inspection is a critical task in ensuring the surface quality of steel plates. Deep neural networks have the potential to achieve excellent inspection accuracy if defect samples are sufficient. Nevertheless, it is very different to collect enough samples using cameras alone. To a certain extent, generative models can alleviate this problem but poor sample quality can greatly affect the final inspection performance. A sample generation method, which employs a generative adversarial network (GAN), is proposed to generate high-quality defect samples for training accurate inspection models. To improve generation quality, we propose a production-and-elimination, two-stage sample generation process by simulating the formation of defects on the surface of steel plates. The production stage learns to generate defects on defect-free background samples, and the elimination stage learns to erase defects on defective samples. By minimizing the differences between the samples at both stages, the proposed model can make generated background samples close to real ones while guiding the generated defect samples to be more realistic. Experimental results show that the proposed method has the ability to generate high-quality samples that can help train powerful inspection models and thereby improve inspection performance.

## 1. Introduction

Steel plates are the industrial intermediate products that are widely used in aerospace, automotive, and other fields [1]. During production and fabrication, various defects appearing on their surfaces is inevitable, which can seriously affect the performance and operation life of the final products. Therefore, surface defect inspection is of great importance in the steel plate production process [2]. Recently, with the rapid development of computer vision and deep learning (DL) techniques, many automatic defect inspection methods based on DL have been proposed and studied for this task, which have the potential to replace the unreliable manual visual inspection in practice [3,4]. However, in most cases, DL methods always suffer from limited training samples due to the difficulty of collecting a large number of defect samples, resulting in poor inspection results.

During production, collecting enough defect images is very hard and time-consuming because the emergence of a defect is very rare and random. The most intuitive way is to obtain new defect samples via generative models. Researchers have adopted many types of generative models in recent years, among which the generative adversarial network (GAN) has attracted the most attention in the field of industrial inspection [5,6]. Unlike typical data augmentation methods [7], GANs can bring in more useful knowledge that benefits model learning through the adversarial training process between the generator and the discriminator. The former only uses knowledge to transform or crop images, which has significant limitations in practice.

Although GANs have achieved encouraging generation performance, there are still several challenges in generating defect samples by GANs. One is that the initial training of a GAN requires a certain number of samples, but in many cases, this may be impossible. The other problem is how to guide the learning direction of a GAN. Different from other generative models, e.g., the auto-encoder, the GAN has no encoding process for input information. It samples directly from the latent space and generates an image through the competition process between its two subnetworks. This means that the feature learning of GANs is uncontrollable. If a GAN has learned a simple feature pattern, it will tend to be lazy and not try to learn the complex ones [8] because, at this point, the image synthesized by the generator is realistic enough to deceive the discriminator. However, the surface defect on steel plate may have different texture structures and patterns, causing a GAN to produce low-quality samples and provide little help in training defect inspection models.

In this paper, we present a sample generation method for industrial defect inspection of steel plate surfaces. The proposed method employs a DCGAN (Deep Convolutional GAN) [9] as the basic generative model architecture. With the aim to synthesize high-quality defect samples, we design a production-and-elimination two-stage sample generation process for the network. Therefore, we name the proposed method PE-GAN. This design idea is inspired by the diffusion-and-denoising process of the Diffusion Model (DM) [10]. The DMs are also types of generative models that have achieved superior image generation, but their sampling speed is very slow and the model architectures are too heavy for real-time applications. As a result, the GAN is still the most suitable sample generation model for industrial defect inspection. To overcome the above-mentioned challenges, the PE-GAN combines the common defect and defect-free background samples into training to ensure its convergence after the initial training process. Defect samples are hard to collect due to the scarcity of a defect, but, on the contrary, the background ones are cheap [11]. In this way, the initial training dataset can be available. To improve the generation quality, we use the aforementioned production-and-elimination method to control the learning direction of the PE-GAN. This is a two-stage learning process, but the PE-GAN uses a single generator network twice to implement the production-and-elimination process. In the production process, the generator learns to manufacture defects on defect-free background samples, which simulates the formation of defects on the surface of steel plates. Then, it learns to erase defect objects on defective samples during the elimination stage, which seems to be the opposite process to the former. Finally, we introduce metric learning into the adversarial learning with the aim of minimizing the distribution differences between the generated background samples and the real background samples. Under such learning guidance, the differences between generated defect samples and real ones can be decreased indirectly, and eventually lead to an improvement in the generation quality. Experimental results show that the PE-GAN can generate high-quality samples that help improve the final inspection accuracy while involved into deep model training.

To summarize, the main contributions of this paper are:

(1) the introduction of the PE-GAN, which can be applied to generate high-quality samples for defect inspection on steel plates;

(2) the proposed production-and-elimination method, which guides the generation learning towards the patterns of real ones via simulating the formation of a surface defect; and

(3) a metric learning loss is added into the adversarial learning of the PE-GAN, as well as a demonstration that the proposed PE-GAN can help inspection models achieve competitive results.

The rest of this paper is organized as follows. Section 2 reviews the relevant work on defect inspection approaches, sample generative models, and defect sample generation methods based on GANs. Section 3 presents the framework and details of the proposed PE-GAN. Section 4 reports the experimental results. Finally, Section 5 concludes this paper.

## 2. Related Works

### 2.1. Defect Inspection Approaches

In the past, a typical defect inspection method includes two parts: a feature extractor and a pattern recognizer. The former is to extract handcraft features such as Haar [12] and Local Binary Pattern (LBP) [13], and the latter, e.g., Support Vector Machine (SVM), is to process and analyze the extracted feature patterns. Thus, the traditional methods are the combination of different feature extractors and pattern recognizers. For instance, Ghorai et al. [14] extracted the set of wavelet features and then fed them into a SVM to identify surface defects. Song et al. [15] used an improved LBP to remove noise and the combination of the Nearest-Neighbor Classifier (NNC) and SVM for defect classification. However, this kind of methods, commonly known as feature engineering, is highly dependent on the design and selection of hand-craft features. It causes poor generalization and universality due to the over-subjective feature design, so the applications of these traditional methods are always limited in the real industrial condition.

Recently, deep learning techniques have achieved remarkable results in many vision detection tasks. Many works have focused on this technique and deep neural networks for industrial defect inspection. The main problem to be solved when using such a model, e.g., the most commonly used convolutional neural network (CNN), is how to train it due to the limited defect samples in industry. The existing methods are mainly based on two schemes. The one is to reduce the size and complexity of a deep neural network, in order to alleviate the hard requirements on the training samples. For example, the works [16,17] designed small scale CNNs and the work in [18] simplified the benchmark CNN models. Nevertheless, this simplification strategy also leads to a decrease in the representability of the model, which usually only applies to the defect datasets with simple patterns. The other scheme utilizes transfer-learning techniques for fine-tuning the pre-trained models.

For instance, Ren et al. [19] used a pre-trained CNN model to perform a defect segmentation task on several industrial production surfaces. He et al. [20] proposed an end-to-end defect inspection method based on the pre-trained Resnet models. Gao et al. [21] proposed a defect recognition method using a pre-trained VGG16 network. This type of method is equivalent to the process of initializing model parameters, and the migrated model trained on the defect dataset is more prone to convergence. This approach can reduce the original sample requirement of the deep model. However, as soon as the difference in the distribution between the target and source domains becomes too large, this leads to the failure of fine-tuning and poor accuracy.

### 2.2. Sample Generative Models

The fine-tuning scheme is to reduce the dependence on the number of original samples, and another way is to expand the original dataset. The typical approach is the data augmentation but which can only simulate defect locations and simple texture changes, and indeed the limitation in practical applications. Although there have been some successful works in defect inspection [22,23], it cannot be guaranteed that their models and samples are suitable for your tasks. Industrial inspection tasks are very complex. For example, although belonging to the same type of defect, there may be differences between different batches. What is worse, the size of a defect dataset is always small. The intra-class difference can be magnified and severely affect the inspection results. Therefore, it is better to use the samples belonging to the same batch for an industrial inspection task. 

Instead of searching for a universal inspection model, more and more works focused on generative models. These models sample from a noise distribution and continuously learn to approximate the given true data distribution, eventually generating new samples that match the data distribution. Typical generation methods such as auto-encoder (AEs) [24], GANs [9], and DMs [10]. An AE is composed of an encoder and a decoder, where the former compresses the input data and the latter decompresses and reconstructs it. It is widely used for text and image generation [25,26]. Although AEs are good at reconstructing regular features, they are difficult to simulate complex and random samples, resulting in generation of blurred and low-quality samples. The DM is a kind of powerful generative model through a two-stage learning process [27]. One is the forward diffusion process, which adds Gaussian noise to the data until it becomes random noise, and the other is the reverse denoising process, which learns to recover the data structure. Despite DM’s strong performance, its industrial applications are limited due to its slow sampling speed and extremely high consumption of computational resources [28]. GANs train through adversarial learning through two subnetworks, a generator and a discriminator, to achieve the Nash equilibrium. In many fields, there are currently many works using a GAN to generate samples, e.g., [29]. The visual effect of images generated by a GAN is good, and its generation function is not limited and can express multiple distributions. The shortcoming of GAN is the lack of directly effective types of learning guidance, resulting in only synthesizing simple feature patterns into the generated samples.

### 2.3. Defect Sample Generation Methods Based on GANs

For data-limited industrial inspection, GANs are the best compromise between network size and generation effect. As a result, there is an increasing number of related studies to perform defect inspection based on GANs. Researchers used the GAN as a generative model and improved their architecture or learning approach for better results. Gao et al. [6] proposed a contextual loss for the GAN and modified the network on a pix2pix pipeline. Niu et al. [11] developed the defect-generation method called surface defect GAN (SDGAN) that employs two diversity control discriminators to generate diversiform defect samples. He et al. [30] proposed a semi-supervised learning framework that based on multi-training of the categorized DCGAN and a residual network, and finally achieved consistent competitive results under different numbers of unlabeled samples. Hao et al. [31] modified the WGAN to generate new defect samples and integrated attention mechanism into the Resnet34 model for, which achieved gratifying defect classification results. Chang et al. [32] improved the GAN and replaced the backbone network with MobileNetV3 for the real-time requirements. Su et al. [33] proposed a fast defect generation method that used skeleton lines to characterize the defect structure and image inpainting network to reconstruct the broken background. Nowadays, a popular trend is to combine multiple generative models to overcome their shortcomings. In [34,35], they used two types of generative models, the AE and the GAN, which means to assume that the latent variables follow a general distribution instead of the Gaussian distribution, resulting in an improvement of the generation performance. Following this idea, the proposed PE-GAN in this work attempts to use the combination of multiple model structures for defect sample generation. In addition, the production-and-elimination process for the generative model is proposed based on this kind of architecture design. In this way, the PE-GAN can guide the learning direction of the generative model towards high-quality defect image synthesis.

## 3. Methodology

**Figure 1:**
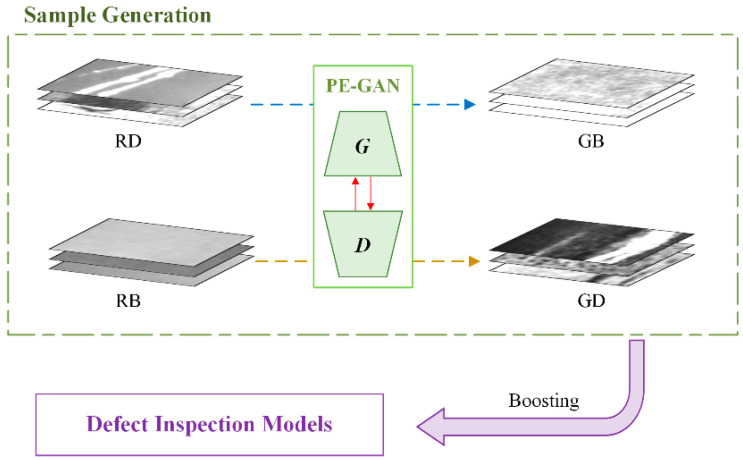
The overview of the proposed method. By the production-and-elimination process based on both the defect and background samples, the PE-GAN can generate a great number of high-quality defect samples to strengthen the defect inspection models. In this figure, the blue dotted line represents the elimination process, and the brown dotted line represents the production process. The notations RD, RB, GD, and GB indicate real defect, real background, generated defect, and generated background samples, respectively.

In this section, we introduce the proposed method PE-GAN in detail. As shown in Figure 1, the proposed method can generate high-quality defect samples through a production-and-elimination learning process. Based on this, a sufficient number of defect datasets, which consist of the real and generated samples, are available. An inspection model can be trained on the dataset and finally perform defect inspection tasks. In Section 3.1, we introduce the network structure of the proposed PE-GAN and the motivation for designing such a network. Section 3.2 details the production-and-elimination learning process and the principle of high-quality sample generation. The loss function and training details of the proposed model are discussed in the Section 3.3.

### 3.1. The Network Framework of the Proposed PE-GAN

For defect sample generation, we adopt the DCGAN model as the basic architecture. In many cases, there are always insufficient defect samples for the initial training of the generative model, because the scarcity of a defect makes it difficult to collect in large quantities. Conversely, defect-free background samples are very easy to obtain in large quantities. Based on this fact, we propose the PE-GAN through modifying the network structure of the DCGAN according to the image-to-image translation paradigm [36]. 

In this way, the PE-GAN can translate image features between different domains at the sample generation stage. To exploit the large number of background samples, the PE-GAN has a production process that generates defect samples from the background domain to a specific defect domain, while converting domains in the opposite way by the elimination process. The high-quality defect samples are generated via mimicking the above process. Further details are discussed in the next section.

**Figure 2:**
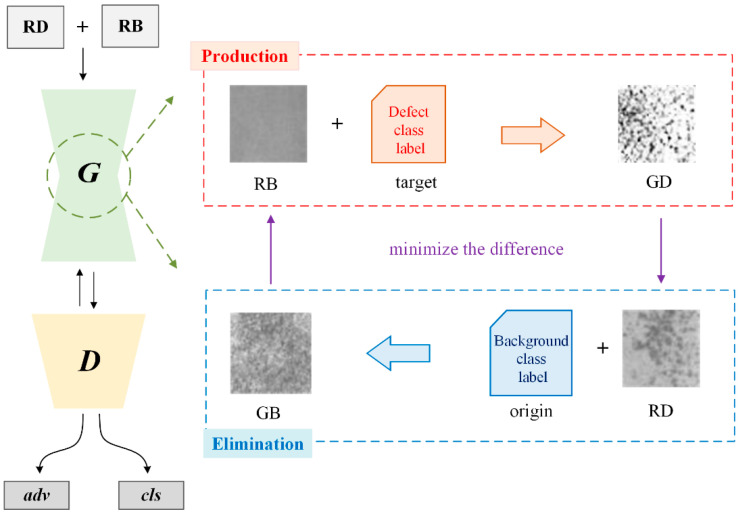
The framework of the proposed PE-GAN. The proposed method combines the RB and RD samples into generation training. In production process, the PE-GAN translates RB to GD with a random defect class label as target; in elimination process, RD is translated to GB under the background class label. By minimizing the difference in both processes, PE-GAN can guide the learning direction and thus generate high-quality defect samples.

The PE-GAN consists of two sub-networks: a generator and a discriminator. Their network architectures are shown in Table 1 and Table 2. For the generator, we modify the original structure of the DCGAN following the popular image-to-image translation network structures [36,37]. Specifically, three convolutional layers (CONV) are first used to down-sample the resolution sizes; then six residual blocks are embedded in the middle of the network for feature representation; and finally, the deconvolution layers (DECONV) are adopted for up-sampling. The activation units ReLUs follow behind each layer except the output layer uses the Tanh function. The instance normalization is also used in the generator as in [38]. For the discriminator, except for the layer parameters, the architecture is the same as the DCGAN. The Leaky ReLU units with a negative slope of 0.01 are used behind convolutional layers to prevent the gradient vanishing. The discriminator has two output layers: the one is the adversarial (adv) output layer which distinguishes whether the output sample is real or synthesized; the other is a classification output layer (cls) over C defect categories plus one background category. The notation w × h is equal to 1/32(W × H), where W × H represents the input size of the discriminator. The output functions are defined and discussed in the following section. The famework of the PE-GAN is shown as in Figure 2.

### 3.2. PE-GAN for Defect Sample Generation

As in [36], we also use the class label as the domain label so that there are *C* defect domain labels and a background domain label in the training of the PE-GAN. As with the typical GANs, the purpose of applying the PE-GAN is to generate realistic defect images by the competition learning of a generator *G* and a discriminator *D*. The *D* learns to distinguish between real and fake samples, while the *G* learns to generate realistic samples to fool the *D*. Therefore, the PE-GAN also needs to introduce the adversarial loss that makes the generated samples close to the real ones as much as possible. The adversarial loss is defined as:

(1) $$\underset{G}{\min}\;\underset{D}{\max}V(G,D)=E_{p_{data}(x)}\log D(x)+E_{p_{z}(z)}\log[1-D(G(z))]$$

where is the empirical estimate of the expected probability values. The *G* transforms the input noise *z* into *G*(*z*), which is sampled from a random noise distribution $p_{z}$, and the ideal $p_{z}$ should converge to the real data distribution $p_{data}$.

**Figure 3:**
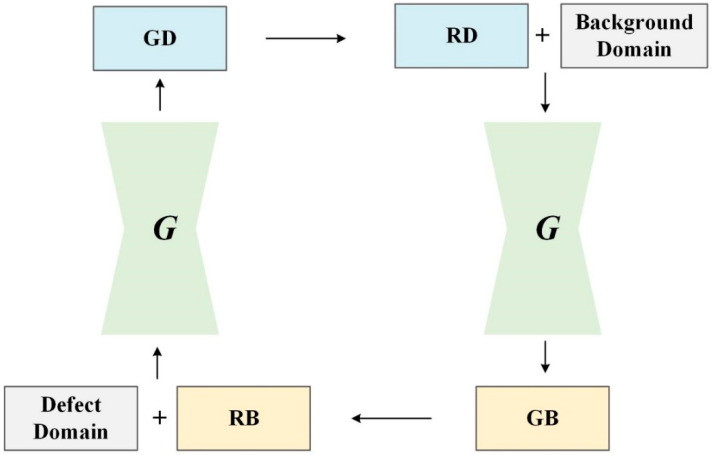
The production-and-elimination generation process. Note that this process uses a single generator twice. The production translates a background sample into a defect image in the selected defect domain and then reconstructs a background sample from the generated defect sample.

However, the generator of the PE-GAN is an image-to-image architecture, whose goal is to learn mappings between defect domains and the background domain. To achieve this, we train the *G* via the proposed two-stage production-and-elimination strategy, as shown in Figure 3, where an input sample is translated into an output one conditioned on the target domain label. In other words, the PE-GAN performs sample image translation using G twice in each training step. Specifically, in the production process a background sample is translated into a fake defect sample by randomly assigning a defect domain label, performing RB→GD; while in the elimination process, a defect sample is translated into a fake background sample, performing RD→GB. The production process can obtain many new defect samples under a large number of background samples, but the generation quality cannot be guaranteed due to the GAN prefers to learn simple patterns. Therefore, the PE-GAN introduces the corresponding elimination process and can improve the generation quality by minimizing the difference either between RB and GB or RD and GD. According to Equation (1), the adversarial loss of our method is rewritten as:

(2) $$L_{adv}=\underset{G}{\min}\;\underset{D_{src}}{\max}V(G,D_{src})=E_{x}\log D_{src}(x)+E_{x,c+1}\log[1-D_{src}\left(G(x,c+1)\right)]$$

where *G* generates the *G*(*x*, *c* + 1) conditioned on the input sample *x* and the target domain label *c* + 1. The *c* + 1 represents *c* defect domain labels plus one background domain label. As usual, *D* tries to distinguish between real and fake samples. The term *D_src_*(*x*) is defined as a probability distribution over sources given by *D*. The *G* and *D* attempts to minimize and maximize this objective, respectively.

**Figure 4:**
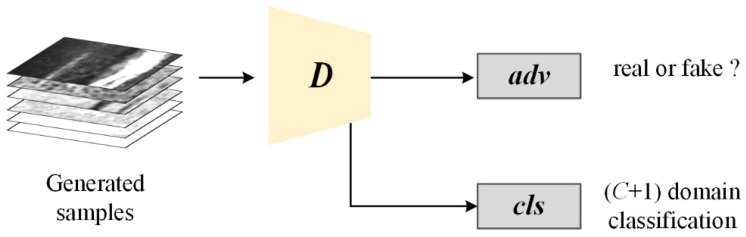
The output of the discriminator.

In order to translate an input sample with a domain label into an output sample, we need to add an additional classification output layer for the *D*. As shown in Figure 4, the *D* of the PE-GAN has two output layers: the one is the original one that distinguishes between real and fake samples, and the other is the (*c* + 1) domain classification layer that assigns the output samples to a proper target domain. The parameters of these layers are given in Table 2.

To achieve the domain assignment, we use the domain classification loss defined in [35], which consists of two parts: The classification loss term of real samples $L_{cls}^{r}$ used for the optimization of *D* and the classification loss term of fake samples $L_{cls}^{f}$ used for the optimization of *G*. They are defined in Equations (3) and (4), respectively.

(3) $$L_{cls}^{r}=E_{x,c^{\prime }+1}\log[-\log D_{cls}(c^{\prime }+1|x)]$$

By minimizing the objective as in Equation (3), *D* learns to classify a real image *x* to its corresponding original defect domain *c*′ or the background domain.

(4) $$L_{cls}^{f}=E_{x,c+1}\log[-\log D_{cls}(c+1|G(x,c+1))]$$

Correspondingly, *G* tries to minimize the objective as in Equation (4) to generate samples that can be classified in the target domain (*c* + 1).

By minimizing the adversarial and classification losses defined in Equations (2)–(4), the proposed PE-GAN can generate defect and background samples and assign them into the correct target domains via the production-and-elimination process. However, this generation approach cannot guarantee the quality of the generated samples. We need to guide the learning direction, so that the translated samples can preserve the content of the origin domain and generate only the details related to the target domain. To solve this problem, we use the classic reconstruction loss in the field of metric learning, which is defined as follows:

(5) $$L_{rec}=E_{x,c+1,c^{\prime }+1}[||x-G(c^{\prime }+1,G(x,c+1))|{|}_{L1}]$$

In Equation (5), we use the *L*1 normalization function for the reconstruction loss.

Combining from the Equations (2)–(5), the overall objective functions for the *G* and *D* are written as:

(6) $$L_{D}=-L_{adv}+\lambda_{cls}^{r}L_{cls}^{r}$$

(7) $$L_{G}=L_{adv}+\lambda_{cls}^{f}L_{cls}^{f}+\lambda_{rec}L_{rec}$$

where $\lambda_{cls}^{r}$, $\lambda_{cls}^{f}$, and $\lambda_{rec}$ are hyper-parameters that represent the weights of the domain classification loss and reconstruction loss, respectively, over the adverisarial loss. In this paper, we empirically set them as 2, 5, and 10, respectively.

### 3.3. Training Implementation

Training the PE-GAN that is to optimize the *G* and *D*, whose loss functions are defined in Equations (6) and (7), respectively. We use the defect class labels as the domain ones that also used as input for the *G*. All the input images are reshaped into128 × 128 and 3 channels for RGB. Based on the experience as in [37], we replace the $\lambda_{adv}$ with the Wasserstein GAN objective with a gradient penalty parameter to 10 [39] for ensuring the stability of the training process. We adopt the Adam optimization algorithm [40] with exponential decay parameters *β*_1_ and *β*_2_ are set to 0.9 and 0.99, respectively. We set a mini-batch size of each past to 16, a learning rate to 0.0001 and total epochs to 20,000.

## 4. Experiments

In this section, we conduct extensive experiments to evaluate the performance of the proposed PE-GAN. In Section 4.1, we describe the defect dataset and evaluation metrics. We assess the quality of the generated defect samples in Section 4.2. Finally, we demonstrate their capacities in training powerful inspection models and improving the defect inspection performance in Section 4.3. All the experiments are conducted on a single NVIDIA RTX 4090 GPU.

### 4.1. Defect Dataset and Metrics

**Figure 5:**
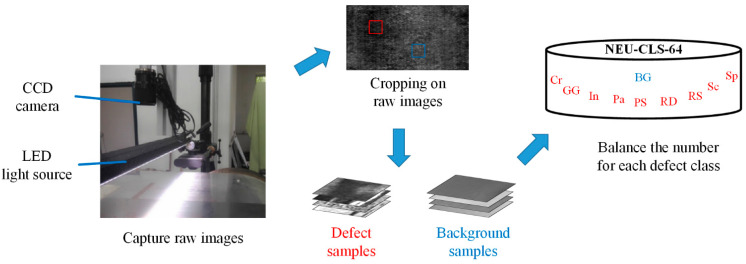
Construction of the NEU-CLS-64 dataset.

We evaluate the proposed PE-GAN on the NEU-CLS-64 defect dataset, where the samples are collected from actual steel plates by CCD cameras. The captured image is usually of high resolution, which is difficult to use directly in training deep models. Not only that, a defect usually occupies only a small part of the raw image. Therefore, we need to crop these raw images and obtain defect-centred samples for training models in an efficient way. A large number of defect-free background samples can now be easily obtained. As shown in Figure 5, the raw images are captured by the industrial CCD camera (Basler, Ahrensburg, Germany, acA640-90uc) under the LED light source (CCS, Tomakomai, Japan, HLND-1200SW2). Next, we obtained a small number of defect samples and a large number of background samples by cropping the raw images. The resolution size of all the samples is 64 × 64. Finally, select the defect samples randomly while ensuring class balance in the dataset.

**Figure 6:**
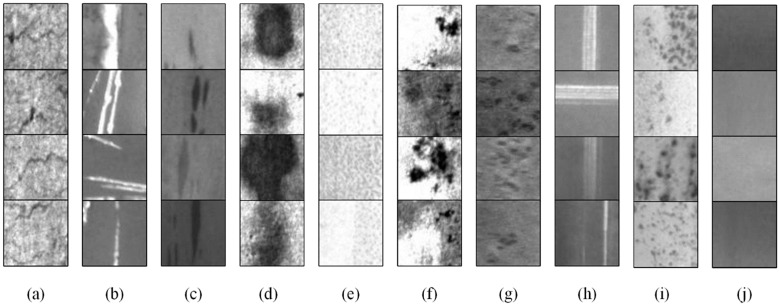
Examples of the samples in the NEU-CLS-64 dataset. (**a**) Cr, (**b**) GG, (**c**) In, (**d**) Pa, (**e**) PS, (**f**) RD, (**g**) RS, (**h**) Sc, (**i**) Sp, and (**j**) background.

There are nine classes of defect samples in this dataset, i.e., crazing (Cr), grooves and gouges (GG), inclusion (In), patches (Pa), pitted surface (PS), rolling dust (RD), rolled-in scale (RS), scratches (Sc), and spots (Sp). The examples are shown in Figure 6. Although there are approximately 7000 samples available in total, the NEU-CLS-64 dataset is imbalanced between classes. We randomly select 200 samples in each class in terms of the class with the fewest samples. In addition, we cropped and collected 1000 background samples from the raw images captured from the actual steel surface. The selected defect samples are divided into training/testing sets in a ratio of 7:3, and all the background samples are added to the training set.

In the following experiments, we employ the Frechet Inception Distance (FID) [41] to evaluate the quality of generated samples. The FID score is the most widely used metric used to evaluate the difference between the generated data distribution and the real data distribution. To achieve it, the FID calculates the Frecheti distance between the two distributions, taking into account both the mean and covariance. The lower the FID, the higher the generation quality that means the synthetic images are more realistic. The function of FID is written as:

(8) $$FID(x,x\prime )=\left\|\mu_{x}-\mu_{x\prime }\right\|+Tr(\Sigma_{x}+\Sigma_{x\prime }-2\sqrt{\Sigma_{x}\Sigma_{x\prime }})$$

where *x* and *x*’ are the real and the generated samples, respectively. The notions *μ* and Σ represent the mean and covariance matrix, and *Tr* represents the trace of a matrix.

To evaluate the performance of the generated samples, we train inspection models on them and perform defect classification tasks. We adopt the commonly used metric of accuracy for the experiments at this stage.

### 4.2. Defect Dataset and Metrics

**Figure 7:**
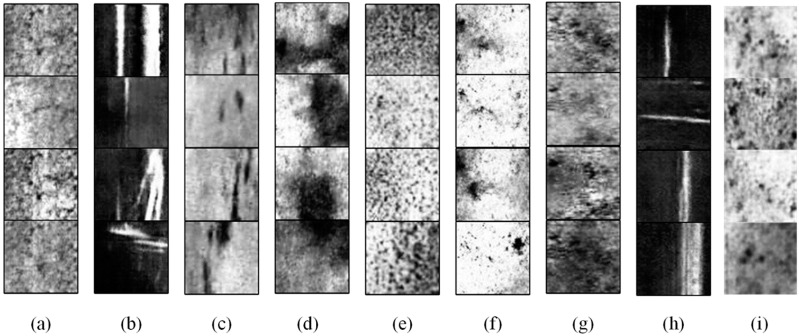
Examples of the generated samples. (**a**) Cr, (**b**) GG, (**c**) In, (**d**) Pa, (**e**) PS, (**f**) RD, (**g**) RS, (**h**) Sc, and (**i**) Sp.

Figure 7 shows the examples of samples generated by the proposed PE-GAN. As in Figure 6, we can observe that most defect classes can generate realistic images that cannot be intuitively distinguished from the real images by the human eye, such as GG, In, Pa, and PS, etc. Unsurprisingly, several classes, such Cr and RS, obtain poor generation results. This is mainly because the features of these defects are too small, which makes it easy to confuse with the surface texture of the steel plate.

The comparison results with other generative methods are shown in Table 3. The comparison methods include DCGAN [9], LSGAN [42], CycleGAN [36], and StarGAN [37]. The selected generative models can be divided into two types. The DCGAN and LSGAN are the classical GAN structures that sample from a random latent space. The CycleGAN and StarGAN are based on the image-to-image translation architecture. As shown in Table 3, the classic generative methods generally seem to obtain less satisfying FID scores. Comparatively, the image-to-image translation methods can achieve a huge improvement on the average FID score. The proposed PE-GAN obtains the lowest average FID score over nine defect classes, which means our method achieve the best results on sample quality comparing to other methods. Although the PE-GAN does not achieve the consistently competitive results on all the categories, it still outperforms other methods in more than half of them. We empirically think the reason is that the PE-GAN has a superior learning guidance, the production-and-elimination process, for sample generation. Our method can learn how to produce a defect on a background sample and how to eliminate a defect on a defect sample. In such a two-stage process, the generator can better learn defect patterns and distinguish between defects and background textures. Therefore, our method achieves the best results through the production-and-elimination process, which demonstrate its superior performance in defect sample generation.

### 4.3. Defect Inspection Results with Generated Samples

In general, industrial inspection should have characteristics of real-time and accuracy. The samples in the NEU-CLS-64 are all the small size. Therefore, we select the ResNet18 [43] and MobileNet [44] models as the defect inspectors in this section. The former is a residual network, which has four residual blocks and a skip connection is used in each block. The latter consists of inverted residual blocks, which have deep convolutional and pointwise convolutional layers. Both models are lightweight, which better fits the actual needs of industrial testing, and are public models, which can be easily obtained from the Internet.

Because of these lightweight model structures, we train models directly on the defect dataset instead of pre-trained models, which can avoid the impact of distribution differences between common objects and industrial defects. We regard none generated samples scheme as the baseline for comparison, where models are trained on the original dataset. Based on the ResNet18 and MobileNet models, we use the generated samples produced by the different generative models mentioned in the previous section. As a successful experience in [5], we include generated samples that are three times more than the real ones, i.e., 600 generated samples for each defect class, into the training. The comparison results for defect inspection are shown in Table 4. From this table, we can see that the accuracy has a great improvement when generated samples are added. The samples generated by image-to-image methods still have a better accuracy improvement effect than those generated by classic methods. It means that the quality of the generated samples plays a crucial role in defect inspection. Our proposed PE-GAN achieves the best inspection results, which proves that these generated samples are of very high quality and thus can improve the performance of the defect inspection model.

## 5. Conclusions

In this paper, we propose a novel generative method PE-GAN for defect inspection of steel plates. Through simulating the formation of defects on the surface of a steel plate, we design the production-and-elimination process in the PE-GAN for defect sample generation. At the production stage, the PE-GAN learns to generate defects on background samples while it learns to erase defects on defect samples at the elimination stage. By this process, the PE-GAN can minimize the differences between the samples at both stages and thus make generated samples close to real ones. Finally, a metric-learning-based loss function is introduced to guide the learning direction of the model for the generation of higher quality and more realistic defect samples. Experimental results can demonstrate the effectiveness of our method. The PE-GAN can generate high-quality defect samples and help to train strong models, and ultimately improve the performances of industrial defect inspection methods.

## Figures and Tables

**Table 1:** The network architecture of the generator.

Type	Filters	Size/Stride	Activation Function
CONV	32	7 × 7/1	ReLU
CONV	64	5 × 5/2	ReLU
CONV	128	5 × 5/2	ReLU
RB	128	3 × 3/1	ReLU
RB	128	3 × 3/1	ReLU
RB	128	3 × 3/1	ReLU
RB	128	3 × 3/1	ReLU
RB	128	3 × 3/1	ReLU
RB	128	3 × 3/1	ReLU
DECONV	64	5 × 5/2	ReLU
DECONV	32	5 × 5/2	ReLU
CONV	3	7 × 7/1	Tanh

**Table 2:** The network architecture of the discriminator.

Type	Filters	Size/Stride	Activation Function
CONV	32	5 × 5/2	Leaky ReLU
CONV	64	5 × 5/2	Leaky ReLU
CONV	128	5 × 5/2	Leaky ReLU
CONV	256	5 × 5/2	Leaky ReLU
CONV	512	5 × 5/2	Leaky ReLU
Output (adv)	1	3 × 3/1	Leaky ReLU
Output (cls)	*c* + 1	W × h/1	Softmax

**Table 3:** Comparison with different sample generation methods in FID scores.

Methods	DCGAN	LSGAN	CycleGAN	StarGAN	PE-GAN
Cr	188.62	176.53	180.12	142.33	120.06
GG	256.00	222.07	205.69	180.34	184.67
In	288.35	291.42	240.16	210.71	192.43
Pa	199.07	162.33	140.07	121.88	117.00
PS	255.81	156.91	186.34	144.32	126.97
RD	300.70	193.65	151.13	134.90	108.45
RS	340.52	223.45	177.79	179.42	180.21
Sc	273.49	253.04	227.88	194.51	200.99
Sp	308.96	240.08	190.20	188.39	147.54
Avg	267.95	213.28	188.82	166.31	153.15

**Table 4:** Comparison results for defect inspection in accuracy.

Inspection Models	Source of Generated Samples	Accuracy (%)
Resnet-18	None	93.24
	DCGAN	96.64
	LSGAN	97.67
	CycleGAN	98.89
	StarGAN	99.12
	PE-GAN	99.79
MobileNet	None	91.26
	DCGAN	95.92
	LSGAN	96.61
	CycleGAN	99.16
	StarGAN	99.29
	PE-GAN	99.75

## Data Availability

The dataset is available at: http://faculty.neu.edu.cn/yunhyan/NEU_surface_defect_database.html.
